# Entertainment Media and Gender Norm Transformation Interventions for Young Women and Girls in Sub-Saharan Africa: A Systematic Review

**DOI:** 10.3390/bs15111596

**Published:** 2025-11-20

**Authors:** William Douglas Evans, Elizabeth A. Larson, Courtney J. McLarnon, Michael Hauer, Marian Marian, Sohail Agha, Rajiv Rimal, Beniamino Cislaghi, Elizabeth Costenbader, Amy Henderson Riley, Helen Wang, Sushmita Mukherjee, Sarah Smith, Claire Hunter Davis, Rebecka Lundgren

**Affiliations:** 1 Milken Institute School of Public Health, The George Washington University, Washington, DC 20037, USA; 2 Center on Gender Equity and Health, University of California, San Diego, CA 92093, USA; ellarson@health.ucsd.edu (E.A.L.); cmclarnon@health.ucsd.edu (C.J.M.); mhauer@health.ucsd.edu (M.H.); mmarian@health.ucsd.edu (M.M.); sas022@health.ucsd.edu (S.S.); chd003@health.ucsd.edu (C.H.D.); rlundgren@health.ucsd.edu (R.L.); 3 Behavioral Insights Lab, Seattle, WA 98136, USA; sohailagha@gmail.com; 4 Department of Health, Behavior and Society, Johns Hopkins Bloomberg School of Public Health, Baltimore, MD 21205, USA; rimal@jhu.edu; 5 Institute for Development Studies, Brighton BN1 9RE, UK; cislaghi.ben@gmail.com; 6 Department of Social Work and Social Administration, School of Social Sciences, Makerere University, Kampala P.O. Box 7062, Uganda; 7 PCI Advisory Private Limited, New Delhi 110020, India; bcostenb@gmail.com (E.C.); smukherjee@pciadvisory.com (S.M.); 8 Population Media Center, South Burlington, VT 05403, USA; ahendersonriley@populationmedia.org; 9 Department of Communication, University at Buffalo, The State University of New York, Buffalo, NY 14260, USA; hwang23@buffalo.edu

**Keywords:** social norms, gender norms, entertainment media, systematic review, sub-Saharan Africa

## Abstract

Adolescent girls and young women are particularly vulnerable to the influence of social and gender norms. This systematic review builds on a broader review of social and gender norms interventions, with the overall aim of identifying and mapping empirical evidence on efforts to improve health and livelihood outcomes of adolescent girls and young women in sub-Saharan Africa. The review examines the strategies, methods, mechanisms of change, and research on the effectiveness of the interventions in the field. We conducted a systematic search of peer-reviewed literature using established PRISMA methods. The sample included 35 articles, which represented 24 distinct interventions—the unit of analysis for this systematic review—that spanned 15 countries across sub-Saharan Africa, with eleven in East Africa, six in West Africa, two in South Africa, and one in the Northern and Central regions. Interventions covered a wide range of outcomes, including sexual and reproductive health, gender-based violence, child early marriage, and other areas. The interventions generally served adolescents and young adults up to age 24. Evaluations included observational, quasi-experimental, and randomized controlled designs. Some interventions included social norms measures, and there was varying evidence of effectiveness (from emerging evidence to demonstrated effectiveness). This review suggests that entertainment media is an effective approach for shifting gender norms, attitudes, and behaviors among adolescent girls and young women. More rigorous intervention research is needed.

## 1. Introduction

Social norms are the unwritten rules and expectations for behavior within a society or group (Cialdini et al., 1991; Riley et al., 2021) They have been recognized as important drivers of social and behavioral change and barriers to change in contexts such as low- and middle-income countries (LMICs). Gender norms refer to a specific subset of social norms that govern perceptions of appropriate behavior based on gender identity, and are learned early in life from parents, other family members, and peers, and reinforced in broader social and institutional contexts (Cislaghi & Heise, 2020; Tenenbaum & Leaper, 2002).

While people of all genders and ages are affected by social and gender norms, adolescent girls and young women ages 10–24 are particularly vulnerable to their influence for several reasons. Physical changes, sexual debut, and social pressures and expectations during adolescence create a unique constellation of opportunities and challenges. Additionally, adolescence is a life stage when young people develop attitudes, capacities, intentions, and agency that shape their life course. During this stage, norms within peer and social reference groups play a central role in influencing their behaviors and long-term health and development (Connell & Pearse, 2014; Lazar, 2014; Nguyen et al., 2019; Weber et al., 2019).

As interest in the role of social and gender norms in shaping health and livelihood outcomes has grown, a wave of interventions—often referred to as norms-shifting interventions—has emerged to address these dynamics (Fleming & Agnew-Brune, 2015). Commonly implemented at the community level or through mass media approaches (Lundgren et al., 2021), these interventions seek to improve health and well-being by both disrupting harmful norms and promoting positive norms. Such interventions benefit from the availability of contextual data to help with tailoring and personalizing content to the population (Lundgren et al., 2021). Although approaches to norms shifting vary, many share common characteristics such as prioritizing changes in social norms over individual attitudes, making new behaviors visible as social proof, and engaging social influencers (such as reference groups) in intervention design, among others (Masur et al., 2021).

Norms-shifting interventions aim to influence both direct participants and the broader community, diffusing new ideas, relationships, and behaviors across networks. Many interventions use organized diffusion—online or in person—where a core group intentionally shares new ideas, attitudes, and behaviors with others. Diffusion is essential for sustainability, enabling interventions to reach a tipping point at which enough community members adopt new norms to drive lasting change (Cialdini et al., 1991; Rogers, 1995).

Norms-shifting interventions are designed to produce intermediate outcomes such as changes in social norms and attitudes—particularly those related to gender equity, increased agency, and the creation of a more supportive normative environment. These changes are often expected to trigger a cascade of desired behavioral changes, such as increased use of modern contraception, healthier timing and spacing of pregnancies, greater uptake of HIV testing and preventive behaviors, reduced gender-based violence and child, early, and forced marriage, and improved economic engagement. Together, these outcomes are expected to lead to lasting impact: sustained improvements in the health and well-being of adolescents and young women at the population level (Muralidharan et al., 2015).

### Entertainment Media Background

Through mass media approaches, including radio, television, print, digital media, and informational campaigns, entertainment media presents a unique opportunity for shifting norms. These approaches often address norms by correcting misperceptions and introducing captivating new narratives and messaging, with the potential for wide reach at scale. These interventions feature trusted voices and relatable narratives, an approach that has proven effective in disseminating messages and fostering social change (Gunther et al., 2006). This type of intervention, often referred to as entertainment-education or edutainment (Frank & Falzone, 2021; Lacayo, 2008; Murphy & Phelps, 2022; Riley et al., 2022; Singhal et al., 2004; Singhal & Rogers, 1999), is increasingly recognized for its effectiveness at addressing social norms at scale, both as a standalone strategy and in combination with other approaches (Nasruddin, 2021; Riley et al., 2020; Sánchez-Páramo & Legovini, 2021).

The broad reach and emotional appeal of entertainment media make it a powerful tool for shaping social norms and influencing behaviors across diverse contexts. Entertainment-based media interventions such as reality TV and magazine content challenge harmful norms by using dramatic storytelling, strong character development, and emotionally engaging narratives, alongside factual and unscripted discussions. With its broad presence across platforms and interactive content, entertainment media holds great promise for fostering positive social norms that benefit adolescent girls and young women (Riley et al., 2020). Narrative persuasion can introduce new perspectives and offer alternative role models for youth. Additionally, while entertainment media may directly engage adolescent girls and young women, broader social norms are shaped by influential figures in their lives. Understanding how different groups respond to these messages can help refine communication strategies and identify when complementary reference group engagement is beneficial. Still, in doing so, it is important to assess potential risks, such as backlash against adolescent girls and young women who adopt new behaviors (Lutkenhaus et al., 2023).

The current systematic review builds on the authors’ earlier, broader review of social and gender norms interventions across modalities and strategies. The overall aim of which was to identify and map empirical evidence on social norms to inform future research and interventions aimed at improving health and livelihood outcomes of adolescent girls and young women in sub-Saharan Africa (Center on Gender Equity and Health, 2025). This analysis narrows the focus on entertainment media-based interventions designed to influence social and gender norms in these same LMICs. The primary research question for this review is: What is the evidence on how entertainment media approaches shift social norms among adolescent girls and young women and their reference groups across outcomes? In particular, we explore the components that make entertainment media approaches effective and the role of reference groups in shaping impact. Through this study, we shed light on best practices, gaps in and the state of the evidence, and opportunities to enhance the effectiveness of interventions this growing field of study.

This systematic review examines how entertainment media approaches have been used to shift social norms to improve sexual and reproductive health and women’s economic empowerment, and decrease gender-based violence and child, early, and forced marriage. We highlight interventions implemented across diverse contexts, with a focus on sub-Saharan Africa. This study examines how various entertainment media approaches—single-channel, multimedia, and those with or without community components—are designed and implemented to influence social norms. It explores the mechanisms behind norm-shifting interventions and highlights the role of reference groups and the duration of interventions. It also investigates how norms-shifting outcomes are measured in entertainment media approaches and synthesizes intervention effectiveness in shifting norms, attitudes and behaviors.

## 2. Materials and Methods

We conducted a systematic search of the published, peer-reviewed literature using all relevant major online research literature databases (specified below) and following widely accepted methods for systematic review (Higgins & Green, 2011).

### 2.1. Search Strategy

We aimed to identify all relevant manuscripts published in health and social science literature that used entertainment media to bring about social and gender norms change in sub-Saharan Africa. We based the review methodology on the widely accepted Preferred Reporting Items for Systematic reviews and Meta-Analyses (PRISMA) methodology, and the previous research by the investigative team (Page et al., 2021). Specifically, we searched the following health, social science, and business databases: PubMed, Web of Science (includes Science Citation Index Expanded, Social Sciences Citation Index, and Arts and Humanities Citation Index), CINAHL, Communication & Mass Media Complete, Embase, Google Scholar, and Gates Open Research.

We selected search terms based on the authors’ experiences in the field and conducting previous reviews, and in consultation with a medical research librarian at the University of California San Diego. We applied the following criteria to conduct the search. Studies were included if they focused on improving the health and well-being of adolescent girls and young women aged 10–24 years residing in low-, middle-, or upper-middle-income countries in sub-Saharan Africa. We focused on this population because they have been the focus of extensive programmatic efforts and research in recent years. Eligible studies examined the relationship between social norms and at least one of the following areas: sexual and reproductive health (specifically family planning and HIV), women’s economic empowerment, gender-based violence, and child, early, and forced marriage. Qualitative, quantitative, and mixed methods evaluations from peer-reviewed journals were included so long as they measured social norms or used common ‘proxies’, such as collective attitudes, for social norms. Studies published in English, French, or Spanish from January 2014 to May 2024 were included, whereas studies in other languages were excluded due to language limitations within the research team. Studies were also excluded if they focused on high-income countries or on low- and middle-income countries outside of sub-Saharan Africa.

We employed the following search keywords and logic in our search strategy (Table 1):

### 2.2. Data Analysis

Citations and abstracts of peer-reviewed articles were uploaded into Covidence for screening and full-text review by the investigative team. Covidence was used to detect and remove duplicate entries, then studies were assessed against inclusion and exclusion criteria through a multi-stage screening process. Titles and abstracts were reviewed, followed by full-text assessments to determine relevance. To ensure consistency and clarity in the screening process among team members, all reviewers initially assessed a sample of ten article titles and abstracts. Following this exercise, two or more reviewers independently screened all titles and abstracts according to the pre-established inclusion and exclusion criteria. During this process, the team used regular meetings to review the inclusion criteria and discuss any emerging questions or concerns and resolve any disagreements. Reasons for exclusion were documented in Covidence.

We implemented a structured data extraction process based on the Johanna Briggs Institute Evidence Synthesis standard (Aromataris, 2025), and used Covidence software (version Extraction 1) for all data review and analysis (Veritas Health Innovation, n.d.). The extraction tool was adapted to capture key details across the literature. Once the literature was cleared for inclusion after full-text review, two team members conducted extraction in Covidence to draw out relevant data from the literature for further analysis, and a third team member reconciled any dissimilarities between the two extractors. The data extraction tool captured key participant characteristics, including demographics (age, gender, marital status, parity, health literacy, education, caste, disability, and socio-economic status). It also documented whether interventions directly targeted adolescent girls and young women or aimed to shift the norms of influential reference groups such as parents, teachers, and community leaders. Contextual factors, including country, urban or rural setting, and intervention level (individual, household, community, or institutional), were also recorded. Additionally, the extraction tool was designed to accommodate both qualitative and quantitative findings, capturing social norms measurement methods, validity and reliability of measures, health and well-being outcomes, as well as lessons learned, challenges, theoretical approaches, and recommendations. Finally, data on the use of multimedia approaches in norms-shifting interventions were also collected.

Following extraction, the data went through multiple rounds of cleaning and refinement, including verifying data, filling in gaps where possible, and incorporating insights from discussions with a study advisory board and validation workshops in Kenya, Nigeria and India. Interventions were the primary unit of analysis, requiring separate datasets to distinguish general studies from intervention-specific evaluations. Initially, data were compiled into a single spreadsheet capturing details from various sources. A second spreadsheet was then created to synthesize evidence at the intervention level, integrating findings across studies and languages to provide a comprehensive view of each intervention. This structured approach enabled the analysis of key research questions, including how entertainment media interventions applied social and behavior change theories, engaged reference groups, and measured changes in norms. It also supported a holistic assessment of intervention effectiveness and implementation trends, enabling an analysis of the distribution of studies across outcome measures.

**Figure 1:**
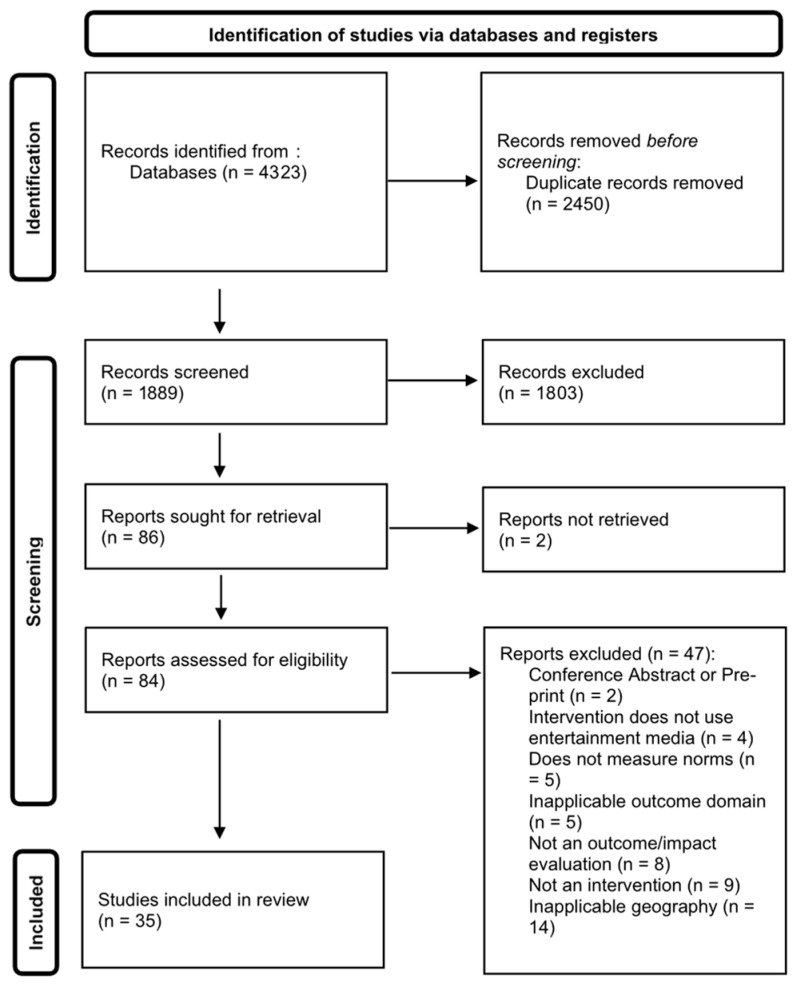
PRISMA diagram.

Figure 1 is the PRISMA diagram summarizing the review and inclusion/exclusion process.

## 3. Results

The sample included 35 articles, which represented 24 distinct interventions—the unit of analysis for this systematic review—that spanned 15 countries across sub-Saharan Africa, with eleven in East Africa, six in West Africa, two in South Africa, and one in the Northern and Central regions. Interventions covered a wide range of outcomes, including sexual and reproductive health, gender-based violence, child early and forced marriage, and other areas. The interventions generally served adolescents and young adults up to age 24, but in some cases, information on the age range of intervention participants was not available. Evaluations included observational, quasi-experimental, and randomized controlled designs (more information is presented below). Some, but not all, interventions included social norms measures, and there was varying evidence of effectiveness, ranging from preliminary or emerging evidence to demonstrated effectiveness. Table 2 summarizes the study sample.

Table 3 provides a summary of the key components of intervention design. Several explicit underlying mechanisms of norm change were described in the interventions. All but one intervention (n = 23) disseminated new ideas, such as emerging normative beliefs, in the community, spread through the media channels noted earlier, and other approaches. The next most frequently cited mechanism (n = 14) was a focus on community-level change rather than individual-level change, meaning that 58% of interventions engaged reference groups or people at multiple levels of the socio-ecological system. Finally, just over a third of interventions (n = 9) rooted social norms shifting efforts in the community by engaging communities or individuals in the planning and implementation of norms-shifting strategies, enabling interventions to improve understanding of context-specific norms and the factors that sustain them. Still, fewer than half of the interventions (n = 11) described any engagement with reference groups, and there were no consistent patterns in the most engaged reference groups among those that did. For example, while community members were the most frequently involved group, less than half of the interventions targeted them as a key reference group. This was followed by health workers, who were engaged by just over a third of the interventions (n = 4), and teachers (n = 3).

Social norms shifting content focused on a wide range of topics, including gender-based violence (n = 14), fertility (n = 10), HIV/STIs (n = 9), and sex (n = 7), among other influential norms like marriage, gender, decision-making, and economic opportunities. Of the 24 interventions, eight explicitly referenced a theory of change or conceptual framework outlining how the intervention was intended to work, almost all of which were drawn from social and behavioral theory (n = 7). Examples include Kincaid et al.’s Ideation Model of Social Behavior Change Interventions (Kincaid, 2000), the Socio-Ecological Model (Stokols, 1996), and the Transtheoretical Model of Behavior Change (J. O. Prochaska & Velicer, 1997; J. Prochaska & Diclemente, 1982). Among the 15 interventions that specified a duration (63%), there was a similar distribution across lengths, with five lasting less than one year, five lasting between one and two years, and five spanning more than 3 years. Table 3 provides details on all variables for each intervention reviewed.

The media-related components of interventions are presented in Table 4. A wide variety of channels were used by interventions, with over half using radio (n = 14). Other channels included television (n = 7), social media (n = 7), and digital media (n = 6). The radio and television interventions were typically multi-episode series aired regularly, or public service announcements. For instance, a family planning mass media campaign in Burkina Faso included 90 s radio spots broadcast ten times per day in addition to three one-hour interactive phone-in radio programs a week to disseminate and engage on the benefits of family planning. Social media posts, print materials, regular text messages, or discussion groups commonly complemented these interventions to increase engagement and information sharing. Just over half of interventions focused on standalone approaches with only one channel (n = 14), with the remaining using a multimedia approach (n = 10). The majority (n = 16) of interventions used in-person components, such as listening and viewing clubs, facilitated community discussions, and direct outreach, while 50% (n = 12) of interventions engaged participants virtually. Notably, only seven interventions (30%) employed both in-person and virtual components.

Table 5 describes the evaluation approaches and main outcomes of interest of the 24 interventions. A variety of intervention designs were employed. Over one-third conducted randomized controlled trials (RCTs) (n = 9) and just under one-third (n = 7) were evaluated via longitudinal cohorts. Other evaluation approaches included quasi-experimental designs (for example, propensity score matching; n = 2), cross-sectional studies (n = 2), and pre-post test designs (n = 1). Of note, only one of the included interventions, *Merci Mon Héros*, employed mixed methods to evaluate outcomes.

We analyzed the sample for the use of social norms measures (either explicit or a proxy, such as collective attitudes) and evidence of a shift in norms associated with the intervention. Of the 24 interventions, 15 reported on the evaluation of norm measures, and all reported clear evidence of effectiveness, emerging evidence, or, in some cases, both for different measures within the same intervention. For example, the *C’est La Vie!* intervention reported effectiveness in changing gender-based violence-related norms and reported emerging evidence for child, early, and forced marriage and family planning-related norms. The most frequently cited effective norms change was for gender-based violence (n = 5), followed by family planning (n = 3). The remaining ten interventions (41%) report no explicit social norms measures.

We also analyzed the sample for explicit measures of attitudes and beliefs, such as an attitude opposed to gender-based violence or in favor of contraceptive use. Of the 24 interventions, 20 reported explicit measures of attitudes and/or beliefs, and all but one, which had unclear evidence, reported clear or emerging evidence of effectiveness, or both. For example, the *Shujaaz* multimedia platform reported effectiveness in changing women’s attitudes toward economic empowerment and emerging evidence on family planning attitudes. The most frequently cited effective attitude change was for gender-based violence (n = 6), followed by family planning (n = 2) and child and early forced marriage (n = 2). The other four interventions reported no explicit measures of attitudes or beliefs.

Finally, we analyzed the sample for explicit behavioral change measures, such as an increase in contraceptive use. Of the 24 interventions, 18 reported explicit behavioral measures and all of these reported some evidence of effectiveness, with two interventions reporting mixed results (ineffective at shifting behaviors around gender-based violence, with emerging evidence on shifts in HIV-related behaviors) and one reporting ineffectiveness for change in gender-based violence behavior alone. Effective interventions demonstrated statistically significant positive shifts in outcomes, while ineffective interventions demonstrated statistically significant negative shifts. Interventions with emerging evidence of effectiveness did not demonstrate a statistically significant change in either direction. Some interventions, such as *Soul Buddyz Clubs*, reported evidence of impact across multiple behaviors, including family planning and HIV. The most frequently cited effective behavioral change was for family planning (n = 5), followed by gender-based violence (n = 4), and HIV (n = 3) The remaining six interventions reported no behavioral measures.

## 4. Discussion

Social norms interventions for adolescent girls and young women using entertainment media in LMIC are a growing area of study, and there is an increasing body of evidence emerging from sub-Saharan Africa. This systematic review identified 24 interventions published in peer-reviewed journals since 2014, conducted across 15 countries in sub-Saharan Africa. In the literature, there is a common belief that norms, attitudes, and behaviors are difficult to change (Batzke & Ernst, 2024). However, all interventions in this review showed clear or emerging evidence of effectiveness in at least one of these areas, and some showed progress in all three. Therefore, this review underscores the importance of such interventions in promoting health and well-being among adolescent girls and young women in sub-Saharan Africa, offering valuable insights into what strategies work and where gaps still exist in norms-shifting efforts. This study represents the first systematic review of evidence on entertainment media interventions to influence social and gender norms in LMIC. It provides a foundation for future research and interventions in the field.

We found that variety of intervention channels and strategies are used in the field. Radio and other mass media, along with community-based and interpersonal approaches, are the most common. Additionally, interventions cover a wide range of topics related to the health and well-being of adolescent girls and young women. While some interventions focus on a single topic, such as HIV or gender-based violence, most approaches are combined and applied across multiple topics and media channels, allowing interventions to reach audiences in different settings, at different times, and through multiple engagement strategies. For example, Shuzaaz, which aimed to improve health and well-being outcomes related to family planning and women’s economic empowerment, used weekly radio shows complemented by printed comics and social media approaches. This expanded the intervention’s reach and led to effective changes in attitude towards women’s economic empowerment and effective behavioral changes related to family planning and women’s economic empowerment.

While reporting on the included interventions was generally thorough, some variables were not always reported. For example, the demographic characteristics of the population served were only available in 14 of the 24 publications. Additionally, most interventions lacked information on engaged reference groups. While this might reflect the broad reach of media-related interventions, making it challenging to target specific groups, it also limits our ability to understand who to focus on for future changes. Additionally, only six of the 24 interventions had an explicit theory of change or conceptual framework. Use of theory is a hallmark of well-designed and effective interventions in most social and behavioral research. Theory not only strengthens intervention design but also enhances the potential for replicability, as it allows for clearer testing of mechanisms of change and contributes to an understanding of what works in shifting social norms. Given that social norms change interventions for adolescent girls and young women in LMICs represent a relatively new and emerging field, it is perhaps unsurprising that theory is not always explicitly or systematically applied. Some interventions may have drawn on theoretical frameworks, but they did not describe these frameworks in published articles. Nonetheless, this represents a crucial area for growth and improvement in the field moving forward.

An important finding is that only three of the 21 interventions explicitly used a digital or social media component (Lutkenhaus et al., 2023), and two more used text messaging. Given the widespread and growing use of mobile phones and the Internet in sub-Saharan Africa, this result is somewhat surprising. The fact that internet penetration has grown rapidly in recent years, during the period of this systematic review (2014 to present), may suggest that the use of digital technologies for interventions targeting adolescent girls and young women is catching up, and that it will become more prevalent in the literature in future years. Furthermore, approaches to addressing diversity and inclusivity were missing from the included interventions. For example, urban/rural inequities in Internet access and how to include those living with disabilities. We argue that it is urgent for future media interventions to address social and gender norms change to utilize digital and social media, especially to reach adolescent and young adult populations. These factors should be examined in future interventions and in reviews of the literature.

This review has several important implications for future entertainment media interventions. First, it highlights the need to develop standards for reporting on interventions and evaluation studies in this field. We found many inconsistencies and gaps in reporting measures, intervention approaches, theoretical frameworks, and participant characteristics—information that will be crucial for future research. Additionally, many aspects of the interventions and their effects on outcomes remain unclear, such as the effectiveness of standalone versus multimedia approaches, the use of in-person and virtual components, as well as dose, frequency, and duration, among other factors. Establishing uniform publication standards and including an assessment of these components will strengthen the evidence base and help advance the field.

Second, there is little information available on the effectiveness of specific intervention strategies (e.g., community dialogue or social media) in multi-component interventions. Isolating which strategies are effective for specific outcomes (e.g., gender-based violence or family planning) in specific country contexts would improve the chance of future intervention success and help to build evidence of effectiveness. This is an issue of both intervention design and aligning evaluation approaches to capture the necessary evidence. This also highlights another dimension of the insufficient reporting that was noted earlier: implementers should be encouraged to provide detailed information on strategies and specific outcomes in future publications.

Third, access to and use of specific entertainment media modalities are undergoing rapid changes. Exposure to social and other digital media is increasing in LMICs, and consumers are increasingly using digital devices to consume entertainment media content. For example, YouTube is increasingly used to view TV programming, podcasts are used to consume audio content, and streaming services are used to watch films. Short video formats on social media are widely used, especially by youth and young adults. Formative research and a clear understanding of adolescent girls and young women’s media use patterns are critical in tailoring and targeting future interventions to maximize exposure and effectiveness in changing social norms, attitudes, beliefs, and behaviors (Hagg et al., 2018). Future studies should examine the interaction and importance of combining community-based, interpersonal, and digital media channels to maximize effectiveness (Evans et al., 2022).

Fourth, the review also has implications for policy and practice. Given that there are emerging effective approaches in the reviewed studies using entertainment media techniques, policymakers should prioritize funding such programs at scale. Future interventions should leverage policies aimed at increasing girls’ and young women’s agency, educational opportunities, and economic empowerment through promotion of these socially beneficial outcomes in entertainment media.

There are some limitations to this study. First, we included only peer-reviewed literature, which may introduce publication bias by excluding unsuccessful interventions. The larger study from which this systematic review derives examined gray literature (e.g., reports, white papers, and other non-peer-reviewed material). However, as these do not conform to the standards for a PRISMA review, they were excluded. Second, although we employed a comprehensive search strategy and a meticulous coding and review system, some articles may have been missed due to variations in keyword inclusion/exclusion in the literature. This is directly related to issues of reporting—such as inconsistent terminology and gaps in authors’ descriptions—and a standardized publication reporting system for social norms interventions would help address this limitation. Finally, our assessment of quality, particularly in areas such as the evidence and use of theory, is limited by the information reported in the reviewed manuscripts. We relied on qualitative assessments, and future studies should examine these and other variables using quantitative methods (e.g., meta-analysis) to build on the evidence base established in this study.

## 5. Conclusions

This review suggests that entertainment media is an effective approach for shifting gender norms, attitudes, and behaviors related to various health and livelihood outcomes among adolescent girls and young women. Future social norms interventions targeting adolescent girls and young women should explicitly and systematically incorporate social and behavioral theories, with an emphasis on how those theoretical models affect social and gender norms, to enhance rigor and effectiveness. Additionally, digital media, including social media, should be strategically integrated, as these are increasingly the preferred platforms for adolescent girls and young women in LMIC. Moreover, to strengthen the evidence for entertainment media-based norm-shifting interventions, more rigorous evaluations using quasi-experimental and, when appropriate, randomized controlled methods are essential. More studies should ultimately provide what we defined in this paper as clear evidence of effectiveness. The evidence base would also benefit from the consistent use of valid and reliable social norms measures and evaluation designs that specifically examine the connection between intervention delivery (such as exposure and engagement) and outcomes relevant to the field. This will further help advance the understanding and importance of norm-shifting strategies in achieving better health and well-being outcomes among adolescent girls and young women in LMICs, particularly in sub-Saharan Africa.

## Figures and Tables

**Table 1:** Search Strategy.

Adolescent Girls and Young Women Adolescent, Young Adult, Young Woman, Young Women
Countries Sub-Saharan Africa, Angola, Benin, Botswana, Burkina Faso, Burundi, Cameroon, Cape Verde, Central African Republic, Chad, Comoros, Ivory Coast, Cote d’Ivoire, Djibouti, Democratic Republic of the Congo, Equatorial Guinea, Eritrea, Ethiopia, Gabon, Gambia, Ghana, Guinea, Guinea-Bissau, Kenya, Lesotho, Liberia, Madagascar, Malawi, Mali, Mauritania, Mauritius, Mozambique, Namibia, Niger, Nigeria, Republic of the Congo, Rwanda, São Tomé and Príncipe, Senegal, Seychelles, Sierra Leone, Somalia, South Africa, Sudan, South Sudan, Swaziland, Eswatini, Togo, Uganda, Tanzania, Zambia, Zimbabwe.
Social Norms Social norm*, descriptive norm*, injunctive norm*, collective norm*, perceived norm*, normative expectation*, empirical expectation*, normative*, norm*-shifting, reference groups, stigma, backlash, gossip, positive deviance, social influence,
Gender Norms Gender norm*
Sexual and Reproductive Health (HIV/Family Planning) Family planning, contraceptive, contraception, child spacing, birth spacing, birth control, fertility, unmet need, pregnant, pregnancy, adolescent pregnancy, unintended pregnancy, demand generation, abortion, breastfeeding, lactational amenorrhea, pregnancy planning, pregnancy prevention, infertility, fertility intention, fertility desire HIV, HIV infections, human immunodeficiency virus, AIDS, acquired immunodeficiency syndrome
Child Marriage Early marriage, child marriage, teen marriage, adolescent marriage, underage marriage, forced marriage, child bride
Gender-Based Violence Rape, domestic violence, battered women, spouse abuse, wife abuse, family violence, gender-based violence, sex offenses, sexual violence, violence against women, intimate partner violence, physical violence, emotional violence, psychological violence, technological violence, human trafficking, witch killings
Women’s Economic Empowerment Economic empowerment, small business, cash transfer, public transfer, micro-credit, income generation, income generating, cooperative, microfinance, loans, microloans, income, income generation, savings, income distribution, family income, women’s self-help, women’s cooperative, self-help group, support group, lending group, microenterprise group, finance, support group
Multimedia Transmedia, cross-media, social media, digital media, television, TV, radio, podcast, video game*, virtual reality, blog, film, newspaper, storytelling, Facebook, YouTube, WhatsApp, Instagram, Twitter,
Other Program, intervention, project, campaign, initiative, cost-effective, cost-efficiency, scale, scale-up, sustain, sustainability, moderation, mediation, implementation, bounded influence

**Table 2:** Overview of interventions.

Intervention	Outcomes	Geography	Target Population	Reference
BRIDGE (Bridge, Redefine, Integrate, Develop, Generate, and Expand) Program; Bridge II Program	FP, HIV	Malawi	Early adolescent girls (10–14); Older adolescent girls (15–17); Young women (17–24)	(Kaufman et al., 2014) Kaufman, M. R., Rimal, R. N., Carrasco, M., Fajobi, O., Soko, A., Limaye, R., & Mkandawire, G. (2014). Using social and behavior change communication to increase HIV testing and condom use: the Malawi BRIDGE Project. AIDS care, 26(sup1), S46–S49.
C’est la Vie!	FP, GBV	Senegal	Not specified	(Dione et al., 2023) Dione, Malick; Heckert, Jessica; Hidrobo, Melissa; Le Port, Agnès; Peterman, Amber; and Seye, Moustapha. 2023. C’est la vie! Mixed impacts of an edutainment television series in West Africa. IFPRI Discussion Paper 2210. Washington, DC: International Food Policy Research Institute (IFPRI). https://doi.org/10.2499/p15738coll2.137017
ChattyCuz	GBV	South Africa	Young adult women (18–24)	(De Filippo et al., 2023) De Filippo, A., Bellatin, P., Tietz, N., Grant, E., Whitefield, A., Nkopane, P., … & Hatcher, A. M. (2023). Effects of digital chatbot on gender attitudes and exposure to intimate partner violence among young women in South Africa. PLOS Digital Health, 2(10), e0000358. https://doi.org/10.1371/journal.pdig.0000358
Communities Care Programme	GBV	DRC	Older adolescent girls (15–17), Young adult women (18–24)	(Glass et al., 2019) Glass, N., Perrin, N., Marsh, M., Clough, A., Desgroppes, A., Kaburu, F., Ross, B., & Read-Hamilton, S. (2019). Effectiveness of the Communities Care programme on change in social norms associated with gender-based violence (GBV) with residents in Intervention compared with control districts in Mogadishu, Somalia. BMJ open, 9(3), e023819. https://doi.org/10.1136/bmjopen-2018-023819
Confiance Totale	FP	Togo	Early adolescent girls (10–14), Older adolescent girls (15–17), Young adult women (18–24)	(Loll et al., 2023) Loll, D., Tokplo, H., Werwie, T. R., Prince-Agbodjan, S., Ouro-Gnao, D., Vondrasek, C., … & Naugle, D. (2023). Evaluation of the Confiance Totale Campaign in Togo: Associations Between Campaign Exposure and Family Planning Outcomes of Interest. Journal of Health Communication, 28(11), 739–756.
Everyday Heroes	GBV	Uganda	Children (0–9); Early adolescent girls (10–14)	(Uysal et al., 2023) Uysal, J., Chitle, P., Akinola, M., Kennedy, C., Tumusiime, R., McCarthy, P., … & Lundgren, R. (2023). Lessons learned from a mixed-method pilot of a norms-shifting social media intervention to reduce teacher-perpetrated school-related gender-based violence in Uganda. Adolescents, 3(2), 199–211.
Family Planning Mass Media Campaign in Burkina Faso	FP, GBV	Burkina Faso	Older adolescent girls (15–17), Young adult women (18–24)	(Glennerster et al., 2021) Rachel Glennerster & Joanna Murray & Victor Pouliquen, 2021. “The Media or the Message? Experimental Evidence on Mass Media and Modern Contraception Uptake in Burkina Faso,” CSAE Working Paper Series 2021-04, Centre for the Study of African Economies, University of Oxford.
InThistoGether (ITG)	FP, HIV	Uganda	Not specified	(Ybarra et al., 2021) Ybarra, M. L., Agaba, E., & Nyemara, N. (2021). A pilot RCT evaluating InThistoGether, an mHealth HIV prevention program for Ugandan youth. AIDS and Behavior, 25(10), 3437–3448. https://doi.org/10.1007/s10461-021-03237-5
Learning Initiative on Norms, Exploitation and Abuse (LINEA)—radio drama entitled “Msichana wa Kati (Girls in the Middle)”	GBV	Tanzania	Children (0–9), Early adolescent girls (10–14), Older adolescent girls (15–17), Young adult women (18–24)	(Pichon et al., 2022) Pichon, M., Carter, D. J., Howard-Merrill, L., Sono, R., Gimunta, V., Rutenge, O., … & Buller, A. M. (2022). A mixed-methods, exploratory, quasi-experimental evaluation of a radio drama intervention to prevent age-disparate transactional sex in Tanzania. Frontiers in Reproductive Health, 4, 1000853.
Let Us Protect Our Future	FP, HIV	South Africa	Early adolescent girls (10–14); Older adolescent girls (15–17); Young women (17–24)	(Jemmott et al., 2015) Jemmott, J. B., Jemmott, L. S., O’Leary, A., Ngwane, Z., Lewis, D. A., Bellamy, S. L., Icard, L. D., Carty, C., Heeren, G. A., Tyler, J. C., & Makiwane, M. B. (2015). HIV/STI risk-reduction Intervention efficacy with South African adolescents over 54 months. Health Psychology, 34(6), 610–621. https://doi.org/10.1037/hea0000140
Mass Media Experiment in Rural Uganda	GBV	Uganda	Older adolescent girls (15–17); Young women (17–24)	(Green et al., 2020) Green, D. P., Wilke, A. M., & Cooper, J. (2020). Countering Violence Against Women by Encouraging Disclosure: A Mass Media Experiment in Rural Uganda. Comparative Political Studies, 53(14), 2283–2320. https://doi.org/10.1177/0010414020912275
Merci Mon Héros	FP	Côte d’Ivoire	Older adolescent girls (15–17); Young women (17–24)	(Silva et al., 2023) Silva, M., Loll, D., Ezouatchi, R., Kassegne, S., Nagbe, R. Y., Babogou, L., Moussa, F., Werwie, T. R., Portillo, E., Adou, D., Vondrasek, C., Rajan, R., & Dougherty, L. (2023). Evaluating a youth-designed sexual and reproductive health mass and social media campaign in Cote d’Ivoire: triangulation of three independent evaluations. Sexual and reproductive health matters, 31(1), 2248748. https://doi.org/10.1080/26410397.2023.2248748
MTV Shuga Down South	FP	South Africa	Early adolescent girls (10–14); Older adolescent girls (15–17); Young women (17–24)	(Kyegombe et al., 2022) “Nambusi Kyegombe, Thembelihle Zuma, Siphesihle Hlongwane, Mxolisi Nhlenyama, Natsayi Chimbindi, Isolde Birdthistle, Sian Floyd, Janet Seeley & Maryam Shahmanesh. (2022) A qualitative exploration of the salience of MTV-Shuga, an edutainment programme, and adolescentsâ€™ engagement with sexual and reproductive health information in rural KwaZulu-Natal, South Africa, Sexual and Reproductive Health Matters, 30:1, 2083809, https://doi.org/10.1080/26410397.2022.2083809”
MTV Shuga Naija	GBV	Nigeria	Not specified	(Hutchinson et al., 2023) Hutchinson, P., Beaudoin, C. E., Meekers, D., Omoluabi, E., & Akinyemi, A. (2023). “ You need to be able to stand up for what is right”: MTV Shuga Naija’s transformative impact on youth attitudes towards sexual violence in Nigeria. Journal of Interpersonal Violence, 40(13–14), 2984–3013.
Nigerian Urban Reproductive Health Initiative	FP	Nigeria	Not specified	(Krenn et al., 2014) Krenn, S., Cobb, L., Babalola, S., Odeku, M., & Kusemiju, B. (2014). Using behavior change communication to lead a comprehensive family planning program: the Nigerian Urban Reproductive Health Initiative. Global Health: Science and Practice, 2(4), 427–443.
Ouro Negro	FP, GBV	Mozambique	Early adolescent girls (10–14); Older adolescent girls (15–17); Young women (17–24)	(Riley et al., 2020) Riley, A. H., Sood, S., & Sani, M. (2020). Narrative persuasion and social norms in entertainment-education: Results from a radio drama in Mozambique. Health Communication.
Saleema	GBV	Sudan	Young women (17–24)	(Evans et al., 2019) Evans, W. D., Donahue, C., Snider, J., Bedri, N., Elhussein, T. A., & Elamin, S. A. (2019). The Saleema initiative in Sudan to abandon female genital mutilation: Outcomes and dose response effects. PLoS One, 14(3), e0213380.
SASA!	HIV, GBV	Uganda	Older adolescent girls (15–17); Young women (17–24)	(Abramsky et al., 2014) Abramsky, T., Devries, K., Kiss, L., Nakuti, J., Kyegombe, N., Starmann, E., … & Watts, C. (2014). Findings from the SASA! Study: a cluster randomized controlled trial to assess the impact of a community mobilization intervention to prevent violence against women and reduce HIV risk in Kampala, Uganda. BMC medicine, 12, 1–17.
Shujaaz multimedia platform	FP, WEE	Kenya	Young adult women (18–24)	(Hutchinson et al., 2018) Hutchinson, P., Mirzoyants, A., & Leyton, A. (2018). Empowering youth for social change through the Shujaaz multimedia platform in Kenya. International Journal of Adolescence and Youth, 24(1), 102–116. https://doi.org/10.1080/02673843.2018.1475287
Soul Buddyz Clubs (SBC)	FP, HIV	South Africa	Early adolescent girls (10–14); Older adolescent girls (15–17)	(Johnson et al., 2018) Johnson, S., Magni, S., Dube, Z., & Goldstein, S. (2018). Extracurricular school-based social change communication program associated with reduced HIV infection among young women in South Africa. Journal of Health Communication, 23(12), 1044–1050. https://doi.org/10.1080/10810730.2018.1544675
Tamapendo	GBV, CEFM	Tanzania	Not specified	(Green et al., 2023) Green, D. P., Groves, D. W., Manda, C., Montano, B., & Rahmani, B. (2023). A Radio Drama’s Effects on Attitudes Toward Early and Forced Marriage: Results From a Field Experiment in Rural Tanzania. Comparative Political Studies, 56(8), 1115–1155. https://doi.org/10.1177/00104140221139385
Tchova Tchova	FP, HIV	Mozambique	Not specified	(Figueroa et al., 2016) Figueroa, M. E., Poppe, P., Carrasco, M., Pinho, M. D., Massingue, F., Tanque, M., & Kwizera, A. (2016). Effectiveness of community dialogue in changing gender and sexual norms for HIV prevention: evaluation of the Tchova Tchova program in Mozambique. Journal of health communication, 21(5), 554–563.
The Kenya Adolescent Reproductive Health project (KARHP)	FP, HIV	Kenya	Early adolescent girls (10–14), Older adolescent girls (15–17), Young adult women (18–24)	(Njue et al., 2015) Njue, C. and Voeten, H. A. C. M. and Ohuma, E. and Looman, C. and Habbema, D. F. and Askew, I., A. J. Mturieditor, 20163208714, English, Journal article, Ghana, 2308-7854 0850-5780, 29, (2, Suppl.), Accra, African Population Studies, (1934–1953), Union for African Population Studies, Findings of an evaluation of community and school-based reproductive health and HIV prevention programs in Kenya., (2015)
ZAZI	HIV, GBV	South Africa	Older adolescent girls (15–17); Young women (17–24)	(Walker, 2021) “Gavin Robert Walker (2021) “Out there it’s YOLO”: Youth perspectives on a mass media HIV- and gender-based violence campaign in South Africa, African Journal of AIDS Research, 20:1, 79–87, https://doi.org/10.2989/16085906.2021.1872666”

**Table 3:** Key intervention design components.

Intervention	Norms Addressed	Theory/ Conceptual Framework	Reference Groups	Intervention Modalities	Mechanisms of Change	Duration
BRIDGE (Bridge, Redefine, Integrate, Develop, Generate, and Expand) Program; Bridge II Program	HIV	Not specified	Not specified	Community Dialogue, Group Discussion, Mass media	Diffuses new ideas	Not specified
C’est la Vie!	Marriage, GBV	Not specified	Community Members, Family	Community Dialogue, Group Discussion, Clubs/Groups, Mass media, Social Media	Diffuses new ideas	<1 year
Chatty Cuz	GBV	Not specified	Not specified	Not specified	Roots norms shifting in the community, Diffuses new ideas,	Not specified
Communities Care Programme	GBV	Not specified	Community Members	Diffusion, Community Dialogue, Group Discussion, Clubs/Groups	Diffuses new ideas	Not specified
Confiance Totale	Fertility	Based on Kincaid et al.’s Ideation Model of SBC interventions (Kincaid et al., 2012)	Health Workers	Mass media	Communities rather than individual change, Norms visible and catalyzes reflection, Diffuses new ideas, Supports change process	<1 year
Everyday Heroes	GBV	Social Norms Theory	Teachers	Community Dialogue, Group Discussion, Social Media	Roots norms shifting in the community, Communities rather than individual change, Norms visible and catalyzes reflection, Diffuses new ideas, Supports change process,	<1 year
Family Planning Mass Media Campaign in Burkina Faso	Fertility, Gender Norms	Not specified	Not specified	Mass media	Roots norms shifting in the community, Communities rather than individual change, Norms visible and catalyzes reflection, Diffuses new ideas	Not specified
InThistoGether (ITG)	Fertility, HIV	Not specified	Not specified	Not specified	Roots norms shifting in the community, Diffuses new ideas	<1 year
Learning Initiative on Norms, Exploitation and Abuse (LINEA)—radio drama entitled “Msichana wa Kati (Girls in the Middle)”	Fertility, Sex, Marriage, GBV,	Not specified	Not specified	Community Dialogue, Group Discussion, Mass media	Roots norms shifting in the community, Communities rather than individual change, Diffuses new ideas	3+ years
Let Us Protect Our Future	Sex, HIV, Decision-Making/Agency	Not specified	Not specified	Community Dialogue, Group Discussion	Communities rather than individual change, Norms visible and catalyzes reflection, Diffuses new ideas	3+ years
Mass Media Experiment in Rural Uganda	Decision-Making/Agency, GBV	Not specified	Community Members, Family		Communities rather than individual change, Diffuses new ideas, Supports change process	Not specified
Merci Mon Héros (MMH)	Fertility	Theory of change	Not specified	Diffusion, Safe Spaces, Community Dialogue, Group Discussion, Mass media, Social Media, Testimonials,	Communities rather than individual change, Diffuses new ideas	1–2 years
MTV Shuga Down South	Fertility, Sex, HIV, GBV	Based on Badura’s Social Learning Theory (Bandura & Hall, 2018) and Sabido’s Theory of Tone in Human Communication (Sabido, 2003)	Not specified	Mass media, Social Media,	Communities rather than individual change, Diffuses new ideas,	<1 year
MTV Shuga Naija	GBV	Theory of Planned Behavior (Ajzen, 1991)	Not specified	Mass media, Social Media	Roots norms shifting in the community, Communities rather than individual change, Norms visible and catalyzes reflection, Supports change process	Not specified
Nigerian Urban Reproductive Health Initiative	Fertility, Systemic Norms	Theory of Change	Community Members, Health Workers, Religious Leaders	Diffusion, Mass media, Social Media	Norms visible and catalyzes reflection, Diffuses new ideas, Supports change process	3+ years
Ouro Negro	GBV	Not specified	Health Workers	Mass media	Communities rather than individual change, Diffuses new ideas	1–2 years
Saleema	GBV	Not specified	Not specified	Community Dialogue, Group Discussion	Communities rather than individual change, Diffuses new ideas	Not specified
SASA!	Sex, Decision-Making/Agency, GBV	Theory of Change	Community Members, Health Workers	Community Dialogue, Group Discussion, Clubs/Groups	Diffuses new ideas	3+ years
Shujaaz multimedia platform	Fertility, Economic Opportunities,	Not specified	Not specified	Digital Media, Social Media	Roots norms shifting in the community, Communities rather than individual change, Diffuses new ideas	Not specified
Soul Buddyz Clubs (SBC)	Fertility, Sex, HIV	Not specified	Teachers	Community Dialogue, Group Discussion	Diffuses new ideas	3+ years
Tamapendo	HIV, Marriage	Not specified	Not specified	Mass media	Diffuses new ideas	Not specified
Tchova	Sex, HIV, GBV, Gender Norms	Not specified	Not specified	Community Dialogue, Group Discussion, Mass media	Roots norms shifting in the community, Communities rather than individual change, Norms visible and catalyzes reflection, Diffuses new ideas, Supports change process	1–2 years
The Kenya Adolescent Reproductive Health project (KARHP)	Fertility, Sex, HIV, GBV	N/A	Teachers	Safe Spaces, Community Dialogue, Group Discussion	Roots norms shifting in the community, Communities rather than individual change, Diffuses new ideas	1–2 years
Zazi	HIV, GBV, Gender Norms	Social and behavior change theory	Peers, Family	Not specified	Diffuses new ideas	Not specified

**Table 4:** Key media components of interventions.

Intervention	Index of Media Exposure	Standalone or Multichannel	In-Person or Virtual
BRIDGE (Bridge, Redefine, Integrate, Develop, Generate, and Expand) Program; Bridge II Program	Not specified	Standalone: Radio	In-Person
C’est la Vie!	26 episodes, lasting approximately 25 min (three episodes in a row every other week) supplemented by facilitated discussion after screenings	Multichannel: Social media; Digital media; Television; Radio; Podcast	Virtual and In-Person
ChattyCuz	WhatsApp Chatbot Conversations	Standalone: Social Media	Virtual
Communities Care Programme	Not specified	Multichannel: Social media; Radio; Theater	In-Person
Confiance Totale	9, 45 s radio public service announcements	Standalone: Radio	Virtual
Everyday Heros	Facebook and WhatsApp discussion forums within peer-teacher networks	Standalone: Radio	Virtual and In-Person
Family Planning Mass Media Campaign in Burkina Faso	90 s spots broadcasted 10 times per day; 3, 1 h interactive phone-in programs a week	Standalone: Radio	Not specified
InThistoGether (ITG)	5–10 daily text messages for 7 weeks	Standalone: Digital Media	Virtual
Learning Initiative on Norms, Exploitation and Abuse (LINEA)—radio drama entitled “Msichana wa Kati (Girls in the Middle)”	39, 15–20 min episodes	Standalone: Radio	In-Person
Let Us Protect Our Future	Comic workbooks with a series of characters and storylines	Standalone: Comic Books	In-Person
Mass Media Experiment in Rural Uganda	Three short video vignettes screened during the intermission at film festivals (4.5–8 min long); 670 total	Standalone: Film	Virtual
Merci Mon Héroes	Short testimonial videos shared on social media and during community events and discussions	Multichannel: Social Media; Television; Radio; Blogs, Storytelling; Digital Media	Virtual and In-Person
MTV Shuga Down South	12, 22 min episodes	Standalone: Television	Virtual and In-Person
MTV Shuga Naija	10, 22 min episodes (re-aired 18 months later), complemented by a radio drama, social media (website, Facebook, Instagram, Twitter); 8-epiosde follow-up television drama	Multichannel: Social media; Television; Radio; Storytelling; Magazine	Virtual and In-Person
Nigerian Urban Reproductive Health Initiative	30 min weekly radio magazine program, supplementary printed material	Multichannel: Television; Radio	Virtual and In-Person
Ouro Negro	2, 22 min radio episode per week	Standalone: Radio	Virtual
Saleema	Not specified	Multichannel: Social marketing, brand identification	Not specified
SASA!	Not specified	Multichannel: Film; Storytelling; Posters; Television; Radio; Information sheets and Picture cards; Community drama	In-Person
Shujaaz multimedia platform	Weekly syndicated radio show, supplemented by print (comics) and social media (Facebook, SMS, Twitter, WhatsApp)	Multichannel: Social media; Digital media; Radio; Comic Book	In-Person
Soul Buddyz Clubs (SBC)	11 season television drama	Standalone: Television	In-Person
Tamapendo	19 h radio drama (abridged screening of a 20-episode radio drama)	Standalone: Radio	Not specified
Tchova Tchova	34, 12 min radio drama; 9 video profiles of Mozambican trendsetters shown on computer laptops during weekly interactive community sessions for 10 weeks	Multichannel: Radio; Storytelling; Pamphlets	In-Person
The Kenya Adolescent Reproductive Health project (KARHP)	Community: 92 Drama presentations, 35 video shows, 18 IEC material distributions; School: 16 drama presentations (school average), 4 video shows (school average)	Multi-channel: Storytelling; Posters; Community theater	In-Person
ZAZI	Not specified	Standalone: Music Videos	Virtual and in-person

**Table 5:** Evaluation approaches and outcomes of interventions.

Intervention	Study Design	Shift in Norms	Shifts in Attitudes	Shifts in Behaviors
BRIDGE (Bridge, Redefine, Integrate, Develop, Generate, and Expand) Program; Bridge II Program	Longitudinal or Cohort	No Norms Measure	No Attitudes Measures	Effective (HIV)
C’est la Vie!	Randomized Control Trial	Effective (GBV); Emerging Evidence (CEFM, FP)	Effective (CEFM, GBV); Emerging Evidence (FP)	Emerging Evidence (HIV); Ineffective (GBV)
ChattyCuz	Randomized Control Trial	No Norms Measure	Effective (GBV)	No Behavioral Measures
Communities Care Programme	Longitudinal or Cohort	Effective (GBV)	Effective (GBV)	Effective (GBV)
Confiance Totale	Cross-Sectional	Effective (FP)	Emerging Evidence (FP)	Emerging Evidence (FP)
Everyday Heroes		Unclear (GBV)	Unclear (GBV)	No Behavioral Measures
Family Planning Mass Media Campaign in Burkina Faso	Randomized Control Trial	Effective (FP)	Effective (FP)	Effective (FP)
InThistoGether (ITG)	Randomized Control Trial	No Norms Measure	No Attitudes Measures	Emerging Evidence (HIV); Ineffective (GBV)
Learning Initiative on Norms, Exploitation and Abuse (LINEA)—radio drama entitled “Msichana wa Kati (Girls in the Middle)”	Pre-Post Test	No Norms Measure	No Attitudes Measures	Effective (GBV)
Let Us Protect Our Future	Randomized Control Trial	Effective (GBV)	Effective (HIV)	Effective (GBV, HIV)
Mass Media Experiment in Rural Uganda	Randomized Control Trial	Effective (GBV)	Emerging Evidence (GBV)	Effective (GBV)
Merci Mon Héros	Mixed Methods, Cross-Sectional	Emerging Evidence (FP)	Emerging Evidence (FP)	Effective (FP)
MTV Shuga Down South	Longitudinal or Cohort	No Norms Measure	Effective (GBV)	Emerging Evidence (GBV)
MTV Shuga Naija	Quasi-Experimental	No Norms Measure	Emerging Evidence (GBV)	Ineffective (GBV)
Nigerian Urban Reproductive Health Initiative	Longitudinal or Cohort	Effective (FP)	Effective (FP)	Effective (FP)
Ouro Negro	Pre- and Post-Test Design	Emerging Evidence (GBV)	Emerging Evidence (GBV)	No Behavioral Measures
Saleema	Quasi-Experimental	Effective (GBV)	Effective (GBV)	No Behavioral Measures
SASA!	Randomized Control Trial	No Norms Measure	Effective (GBV)	Emerging Evidence (GBV)
Shujaaz multimedia platform	Longitudinal or Cohort	No Norms Measure	Effective (WEE); Emerging Evidence (FP)	Effective (FP, WEE)
Soul Buddyz Clubs (SBC)	Longitudinal or Cohort	No Norms Measure	Emerging Evidence (FP)	Effective (FP); Emerging Evidence (HIV)
Tamapendo	Randomized Control Trial	Emerging Evidence (CEFM)	Effective (CEFM)	No Behavioral Measures
Tchova Tchova	Post-Test	Effective (HIV)	Effective (HIV)	Effective (HIV)
The Kenya Adolescent Reproductive Health project (KARHP)	Randomized Control Trial	No Norms Measure	No Attitudes Measures	Emerging Evidence (HIV)
ZAZI	Longitudinal or Cohort	Emerging Evidence (GBV, HIV)	Emerging Evidence (GBV, HIV)	No Behavioral Measures

## Data Availability

No new data were created or analyzed in this study. Data sharing is not applicable to this article.

## Abbreviations

The following abbreviations are used in this manuscript:

LMIC	Low- and Middle-Income Countries
PRISMA	Preferred Reporting Items for Systematic reviews and Meta-Analyses
